# Propolis-Based Nanostructured Lipid Carriers for α-Mangostin Delivery: Formulation, Characterization, and In Vitro Antioxidant Activity Evaluation

**DOI:** 10.3390/molecules28166057

**Published:** 2023-08-15

**Authors:** Cecep Suhandi, Gofarana Wilar, Ronny Lesmana, Felix Zulhendri, Ine Suharyani, Nurhasni Hasan, Nasrul Wathoni

**Affiliations:** 1 Department of Pharmaceutics and Pharmaceutical Technology, Faculty of Pharmacy, Universitas Padjadjaran, Sumedang 45363, Indonesia; cecep17001@mail.unpad.ac.id (C.S.); ine18001@mail.unpad.ac.id (I.S.); 2 Department of Pharmacology and Clinical Pharmacy, Faculty of Pharmacy, Universitas Padjadjaran, Sumedang 45363, Indonesia; g.wilar@unpad.ac.id; 3 Physiology Division, Department of Biomedical Science, Faculty of Medicine, Universitas Padjadjaran, Sumedang 45363, Indonesia; ronny@unpad.ac.id; 4 Biological Activity Division, Central Laboratory, Universitas Padjadjaran, Sumedang 45363, Indonesia; 5 Kebun Efi, Kabanjahe 22171, Indonesia; felix.zulhendri@kebunefi.com; 6 Department of Pharmacy, Sekolah Tinggi Farmasi Muhammadiyah Cirebon, Cirebon 45153, Indonesia; 7 Department of Pharmacy Science and Technology, Faculty of Pharmacy, Universitas Hasanuddin, Makassar 90245, Indonesia; nurhasni.hasan@unhas.ac.id

**Keywords:** α-mangostin, propolis, nanostructured lipid carrier, antioxidant

## Abstract

α-Mangostin (a xanthone derivative found in the pericarp of *Garcinia mangostana* L.) and propolis extract (which is rich in flavonoids and phenols) are known for their antioxidant properties, making them potential supplements for the treatment of oxidative stress-related conditions. However, these two potential substances have the same primary drawback, which is low solubility in water. The low water solubility of α-mangostin and propolis can be overcome by utilizing nanotechnology approaches. In this study, a propolis-based nanostructured lipid carrier (NLC) system was formulated to enhance the delivery of α-mangostin. The aim of this study was to characterize the formulation and investigate its influence on the antioxidant activity of α-mangostin. The results showed that both unloaded propolis-based NLC (NLC-P) and α-mangostin-loaded propolis-based NLC (NLC-P-α-M) had nanoscale particle sizes (72.7 ± 1.082 nm and 80.3 ± 1.015 nm, respectively), neutral surface zeta potential (ranging between +10 mV and −10 mV), and good particle size distribution (indicated by a polydispersity index of <0.3). The NLC-P-α-M exhibited good entrapment efficiency of 87.972 ± 0.246%. Dissolution testing indicated a ~13-fold increase in the solubility of α-mangostin compared to α-mangostin powder alone. The incorporation into the propolis-based NLC system correlated well with the enhanced antioxidant activity of α-mangostin (*p* < 0.01) compared to NLC-P and α-mangostin alone. Therefore, the modification of the delivery system by incorporating α-mangostin into the propolis-based NLC overcomes the physicochemical challenges of α-mangostin while enhancing its antioxidant effectiveness.

## 1. Introduction

α-Mangostin is a highly significant bioactive compound derived from the pericarp of *Garcinia mangostana* L., commonly known as the mangosteen fruit [1,2]. It boasts a wide range of pharmacological properties that make it an intriguing substance of interest. One of its most notable attributes is its ability to exhibit strong antioxidant activity, effectively regulating the presence of free radicals within the body [3,4]. This remarkable antioxidative capacity can be attributed to three primary mechanisms employed by α-mangostin: direct scavenging of reactive oxygen species (ROS), a hydrogen atom transfer (HAT) mechanism, and activation of the Nrf2 pathway, which ultimately enhances the body’s innate antioxidant defenses [5,6,7]. These inherent antioxidant properties hold immense potential in addressing numerous pathological conditions linked to ROS imbalance. The extensive range of ailments that can benefit from α-mangostin’s antioxidative effects includes cancer, cardiovascular disorders, nervous system disorders, respiratory disorders, rheumatoid arthritis, kidney damage, and sexual maturation [8]. Despite its impressive capabilities as an antioxidant, α-mangostin faces a significant hurdle due to its unfavorable physicochemical properties. These limitations must be carefully considered in the context of its potential applications and further exploration of its therapeutic uses.

α-Mangostin faces challenges related to its solubility in different solvents. While it exhibits good solubility in ethanol and acetone, it is practically insoluble in water [9]. This limited solubility in water poses a significant hurdle in terms of its absorption into the bloodstream, particularly when administered orally [10,11]. The poor absorption rate of α-mangostin consequently leads to low bioavailability [12]. As a result, the potential antioxidant activity of α-mangostin may be compromised due to these physicochemical limitations. To overcome these challenges and maximize the compound’s therapeutic potential, nanotechnology approaches can be employed. By utilizing nanotechnology, it becomes possible to address the deficiencies in the physicochemical characteristics of α-mangostin. This approach offers the opportunity to enhance its solubility, improve its absorption, and ultimately overcome the limitations associated with its current form [13]. Through innovative nanotechnology techniques, researchers can explore ways to optimize α-mangostin’s physicochemical properties and unlock its full antioxidant potential, opening doors to more effective therapeutic applications.

The utilization of lipid-based nanoformulation has proven to be an effective strategy for enhancing the pharmacokinetic characteristics of lipophilic drug compounds. Within the realm of lipid-based nanoparticulate delivery systems, three main categories exist: liquid emulsion (LE), solid lipid nanoparticle (SLN), and nanostructured lipid carrier (NLC) [14]. Among these systems, NLC stands as the most recent and groundbreaking advancement in lipid-based nanotechnology. The implementation of NLC offers several advantages, including improved absorption capacity, enhanced stability, and reduced reliance on surfactant components [15,16]. With the utilization of NLC, a diverse range of components can be incorporated, extending beyond the mere absorption of active compounds [17]. By harnessing the advantages offered by NLC, researchers can explore novel formulations and optimize the delivery of lipophilic drugs, ultimately enhancing their therapeutic efficacy.

In a recent study conducted by Elkhateeb et al., it was discovered that propolis can serve as a valuable component within the lipid phase of the NLC system [18]. Propolis, a natural product, is renowned for its remarkable antioxidant activity owing to its abundant flavonoids and phenolic compounds [19,20]. Both groups of compounds possess the ability to directly scavenge reactive oxygen species, thereby exhibiting potent antioxidant capabilities [21]. The research also demonstrated that the incorporation of concentrated propolis extract into the NLC system further enhances its antioxidant activity [18]. As a result, a propolis-based NLC system holds great promise as a potential solution to overcome the limitations associated with α-mangostin compound while simultaneously augmenting its antioxidant effectiveness. By harnessing the synergistic properties of propolis and the advantages offered by the NLC system, researchers can explore innovative formulations that optimize the therapeutic potential of α-mangostin. This approach not only addresses the physicochemical challenges faced by α-mangostin but also enhances its antioxidant properties, presenting exciting possibilities for future therapeutic applications.

Based on the background presented, this study proposes a propolis-based NLC delivery system for α-mangostin compound. The objective of this research is to develop a propolis-based NLC formulation for α-mangostin delivery, followed by characterization and evaluation of its influence on the in vitro antioxidant activity. Through this study, it is hoped to achieve a new breakthrough in the application of potential natural isolates at more effective dosages.

## 2. Results

### 2.1. Propolis Extraction

The extract yield acquired through the method of maceration employed in propolis extraction is represented in Table 1. The yielded extract amounts to 48.24% (*w*/*w*). It demonstrated compliance with the predetermined standard stipulated for propolis extract derived from the extraction of propolis powder, wherein the minimum required yield is 11% [22].

#### 2.1.1. Phytochemical Screening of Propolis Extract

**Figure 1:**
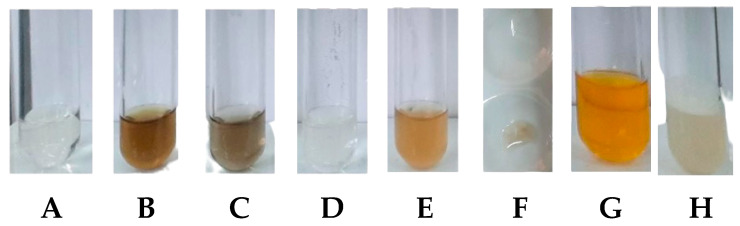
Phytochemical screening results of propolis extract (**A**) using reagents, including FeCl_3_ 5% (**B**), FeCl_3_ 1% (**C**), concentrated HCl + Mg (**D**), NaOH 10% (**E**), concentrated H_2_SO_4_ + acetic anhydride (**F**), and Dragendorff (**G**), and using physical treatment of agitation followed by heating (**H**).

The phytochemical content in the propolis extract was analyzed using various reagents to detect the presence of various secondary metabolite compounds. The visual changes in the extract after each treatment are illustrated in Figure 1. The interpretation of the test results for the content of secondary metabolite compounds is provided in Table 2. The propolis extract used was confirmed to have positive content of phenolic compounds, tannins, flavonoids, and triterpenoids. However, the extract did not show the presence of steroid and alkaloid compounds.

#### 2.1.2. Measurement of Total Flavonoid Levels of Propolis Extract

The total flavonoid content obtained from the propolis extract used is presented in Table 1. The propolis extract was confirmed to contain a total flavonoid content equivalent to 0.39% (*w*/*w*) of quercetin. This result met the standard requirements for propolis extract, which mandates a minimum total flavonoid content of 0.25% [22].

#### 2.1.3. Measurement of Total Phenol Levels of Propolis Extract

The total phenolic content obtained from the propolis extract used is indicated in Table 1. The results showed that the extract contains a phenolic content equivalent to 2.43% (*w*/*w*) gallic acid. This result met the standard requirements for propolis extract, which mandates a minimum total phenolic content of 0.50% [22].

### 2.2. Characterization of Propolis-Based NLC Loaded with α-Mangostin

The prepared NLC formulation was characterized, including particle size, zeta potential, polydispersity index, morphology, entrapment efficiency, and dissolution performance. Each evaluation was conducted according to its specific purpose to confirm the compatibility of the formulated product with the predetermined requirements.

#### 2.2.1. Particle Size Analysis (PSA), Zeta Potential (ZP), and Polydispersity Index (PI)

Table 1 summarizes the obtained values for particle size, zeta potential, and polydispersity index of the unloaded NLC formulation (NLC-P) and the α-mangostin-loaded propolis-based NLC formulation (NLC-P-α-M). The evaluation results showed that NLC-P particles have a size of 72.7 ± 1.082 nm while NLC-P-α-M particles fall within the size range of 80.3 ± 1.015 nm. These results met the size requirements for nanoscale drug delivery systems (1–100 nm) [23].

The zeta potential (ZP) of NLC-P formulation is −5.100 ± 0.954 mV, while for NLC-P-α-M it is −2.767 ± 0.874 mV. These ZP values refer to the surface charge of the formed particles. The obtained ZP values were in line with the use of polysorbate 80 (Tween 80^®^), which has a relatively neutral charge. Particles in the formulation were considered to have a neutral charge when the ZP value falls between +10 mV and −10 mV [24].

The polydispersity index (PI), which indicates the distribution of particle sizes, also showed appropriate results. NLC-P formulation exhibits a PI of 0.211 ± 0.028, while NLC-P-α-M has a value of 0.231 ± 0.028. These results indicated that the particles have a homogeneous size distribution based on the criteria that define nanoformulations with a polydispersity index below 0.3 as having a homogeneous particle size distribution [25].

#### 2.2.2. Transmission Electron Microscopy (TEM) Imaging

**Figure 2:**
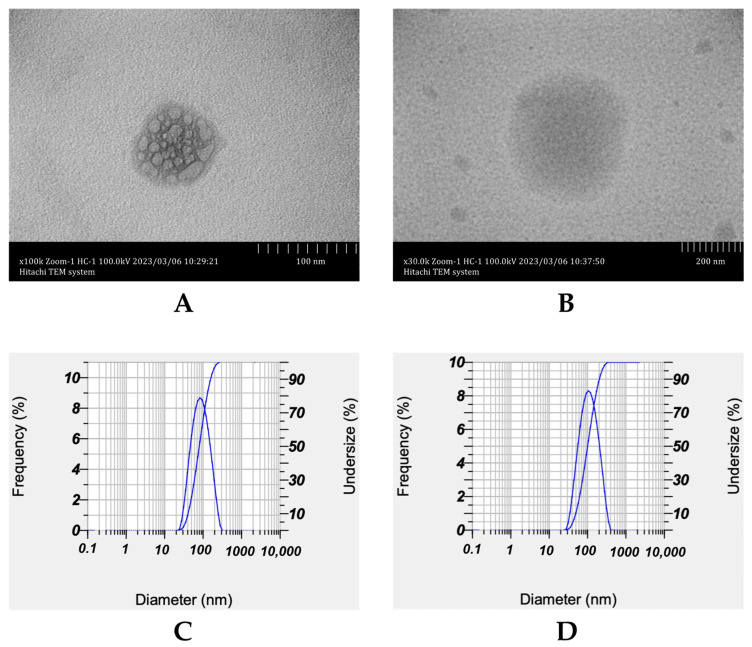
Morphology of NLC-P (**A**) and NLC-P-α-M (**B**); calculated through transmission electron microscopy (TEM) imaging and size distribution of NLC-P (**C**) and NLC-P-α-M (**D**); size distribution was obtained via analysis with a particle size analyzer (PSA).

The morphology of NLC-P and NLC-P-α-M particles was visualized through TEM, as shown in Figure 2A and 2B, respectively. Both NLC-P and NLC-P-α-M particles exhibit spherical morphology. The imaging revealed the presence of lipids within the particles that were encapsulated by surfactants on the surface. These findings were consistent with the characteristics of NLCs, which typically have a spherical shape and lipid content enclosed within the particles [18]. In addition, the particle size distribution of NLC-P and NLC-P-α-M (Figure 2C and 2D, respectively) showed a normal distribution based on data obtained from particle size analysis (PSA).

#### 2.2.3. Entrapment Efficiency

The entrapment efficiency (EE) of the α-mangostin-loaded propolis-based NLC (NLC-P-α-M) is presented in Table 3. The results demonstrated that α-mangostin was successfully entrapped within the propolis-based NLC with a percentage of 87.972 ± 0.246%. These findings indicated the high capability of NLC in effectively encapsulating α-mangostin as a drug compound.

#### 2.2.4. Dissolution Test

**Figure 3:**
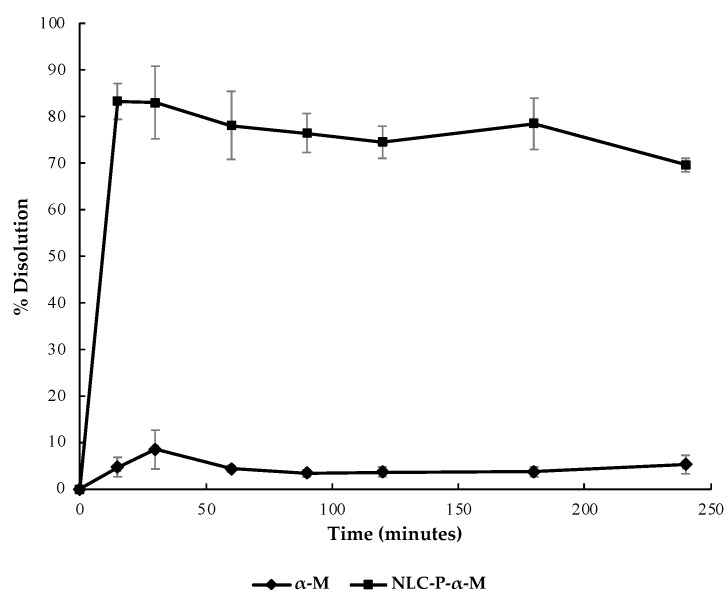
Dissolution of α-mangostin and propolis-based NLC of α-mangostin at pH 7.4.

The dissolution profile of NLC-P-α-M, compared to α-mangostin powder, is illustrated in Figure 3. NLC-P-α-M showed a dissolution percentage of 78.86 ± 3.78% at the 15th minute, whereas α-mangostin powder only reached a dissolution percentage of 8.49 ± 4.17% at the 30th minute. By the 240th minute, NLC-P-α-M had dissolved by 69.33 ± 1.40% while α-mangostin powder had dissolved by 5.23 ± 2.00%. These results indicated an approximately 13-fold increase in the solubility of α-mangostin in the propolis-based NLC system.

### 2.3. In Vitro Antioxidant Activity Assay of Propolis-Based NLC Formulations

**Figure 4:**
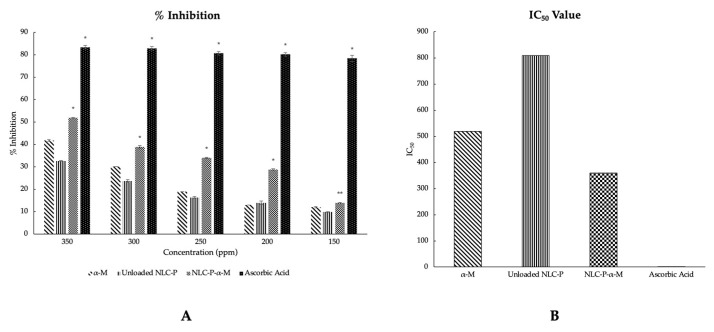
%Inhibition (**A**) and IC_50_ (**B**) comparation of α-mangostin, unloaded propolis-based NLC, and propolis-based NLC of α-mangostin compared to ascorbic acid as the positive control. (*) represents a statistical significance level of *p* < 0.01, and (**) represents a statistical significance level of *p* < 0.05.

The antioxidant activity of the prepared formulations was evaluated based on in vitro testing using the DPPH scavenging assay method. The evaluation results were presented by the percentage inhibition values obtained from each formulation at various concentrations (ppm), as depicted in Figure 4A. Visually, NLC-P-α-M exhibited a heightened inhibitory percentage in terms of antioxidant activity compared to both α-mangostin alone and NLC-P. The percentage inhibition values obtained at each concentration showed significant differences between NLC-P-α-M and both NLC-P and α-mangostin alone. The differences were statistically significant; there was a confidence interval of 0.01 (*p* < 0.01) for almost all concentration variations, except at a low concentration (150 ppm) where there was only a statistically significant difference at a confidence interval of 0.05 (*p* < 0.05). However, the percentage inhibition obtained from NLC-P-α-M was not superior to ascorbic acid as the positive control (*p* < 0.01).

In addition, Figure 4B also explains the IC_50_ values of each formulation. In alignment with the acquired inhibition percentages, the IC_50_ value of NLC-P-α-M demonstrated a decrease in comparison to that of α-mangostin alone and NLC-P. NLC-P-α-M had an IC_50_ value of 359.223 ppm, while α-mangostin alone and NLC-P had IC_50_ values of 519.292 and 809.118 ppm, respectively. However, the IC_50_ value of NLC-P-α-M was still higher compared to ascorbic acid as the positive control (1.297 ppm).

## 3. Discussion

α-Mangostin, the most abundant xanthone derivative in the pericarp of *Garcinia mangostana* L., has been proven to possess excellent antioxidant activity [26]. Previous studies examining collagen-induced arthritis in DBA/1J mice have demonstrated that α-mangostin exhibits a favorable antioxidant profile by reducing the levels of lipid peroxidation end products (NOx and MDA) and maintaining optimal GSH levels [27]. In its development, α-mangostin has been modified into various forms of nanocarriers. These include the use of various nanoparticle technologies, including polymerics, micelles, liposomes, solid lipid nanoparticles (SLN), nanofibers, nanometals, and nanoemulsions. Among the many technological options that have been developed, the development of lipid-based nanocarriers for α-mangostin is the best option considering the high lipophilicity of α-mangostin. Apart from liposomes and SLN, an interesting study conducted by Samprasit et al. found that α-mangostin can also be loaded into the nanostructured lipid carriers (NLC) system, while this study showed that the NLC system exhibited superiority over other nanolipid carrier systems, which can contain multiple components [28]. This study showed that a combination of α-mangostin and resveratrol encapsulated in NLC resulted in a synergistic effect, enhancing the antioxidant effectiveness compared to the individual components alone [29]. In the context of this research, the role of resveratrol was replaced by the antioxidant content of propolis extract. The utilization of propolis extract is advantageous due to its proven antioxidant properties, which can be attributed to its flavonoid and phenolic content. Furthermore, the presence of wax, which has a solid consistency at room temperature, has been demonstrated to contribute to the solid lipid phase formation in NLC systems [18]. Thus, the use of propolis extract can minimize the reliance on lipids, particularly the solid lipid phase, in the production of NLC formulations.

As an active ingredient and supporting component, the propolis extract used must meet established standards. Referring to the standardization proposal for quality control of propolis extracts commercialized in Brazil, propolis extract is considered to have good quality if it has a minimum yield of 11%, a minimum total flavonoid content of 0.25%, and a minimum total phenolic content of 0.50% [22]. In this study, the propolis extract obtained through maceration using 95% ethanol yielded 48.24%, with a total flavonoid content of 0.39% and a total phenolic content of 2.43%. The higher yield was achieved because the use of ethanol as a solvent effectively extracted the active compounds from the propolis powder. These results are consistent when compared to a similar extraction procedure conducted in another study using 70% ethanol as the solvent, which resulted in an extract with a yield of approximately 55% [30]. In addition to flavonoids and phenols, other studies have also confirmed the presence of tannins, triterpenoids, and saponins [31]. Similar findings were obtained from the phytochemical screening of the propolis extract used in this research.

In this study, the NLC system was chosen due to various pieces of evidence suggesting its superiority over other nanoscale drug delivery systems. As a nanolipid carrier, NLC is capable of loading lipophilic active compounds [14]. Additionally, NLC exhibits a higher drug loading capacity compared to other nanoscale lipid carriers such as solid lipid nanoparticles (SLN) [32]. The selection of materials and composition in the production of propolis-based NLC α-mangostin (NLC-P-α-M) was based on evidence from similar studies [33]. Lecithin, as the liquid lipid phase, offers advantages such as low cost, a non-hazardous nature, and the ability to enhance drug permeability [34]. Polysorbate 80 (Tween 80^®^) was chosen as the surfactant due to its high emulsification capacity for lipid components and its non-irritating properties, particularly for topical applications [35]. Furthermore, the melt–emulsification method combined with ultrasonication was selected as it is more environmentally friendly, eliminating the need for organic solvents [36,37].

The NLC formulation obtained in this study has a nanoscale size, a zeta potential close to neutral on its surface, and exhibits uniform particle size distribution, which is indicated by a polydispersity index below 0.3. The negative zeta potential, albeit above −10 mV, indicates a stable neutrally charged surface due to the presence of nonionic polysorbate 80 [38]. The particle size of the NLC obtained was not smaller than similar formulations in other studies. In a study conducted by Elkhateeb et al., NLC-P obtained using the emulsion evaporation–solidification method had a size of approximately 41.57 ± 1.96 nm [18], while in this study the produced NLC-P had a particle size of about 72.7 ± 1.082 nm. This difference may be attributed to the variation in the methods used, particularly the use of an organic solvent (ethanol) in the lipid phase dissolution, which can aid in dissolving the starting materials by molecular or nano dispersion. Additionally, the particle size of NLC-P-α-M is larger compared to the particle size of NLC-P. This increase in particle size is due to the incorporation of α-mangostin, which is dissolved in the lipid component in the NLC system. This also indicates the successful loading of α-mangostin into the NLC system.

The imaging results using TEM also support the presence of α-mangostin in the NLC system. Both NLC-P-α-M and NLC-P particles exhibit a spherical morphology with lipid globules enclosed by the surface of polysorbate 80. The particle size of NLC-P-α-M is relatively larger than that of NLC-P. Additionally, the size of lipid globules in NLC-P-α-M is relatively smaller than in NLC-P. This indicates the dispersion of lipid globules in NLC-P-α-M due to the incorporation of α-mangostin into the lipid phase. However, since α-mangostin is not visible to the naked eye under TEM, the quantitative presence of α-mangostin was measured based on its absorption efficiency. The analysis results showed that approximately 87.972 ± 0.246% of α-mangostin was successfully absorbed into the NLC system. These results are comparable to the entrapment efficiency of α-mangostin in SLN with a drug-to-lipid ratio of 1:2, which yielded an entrapment efficiency of 79.4 ± 2.11% [39]. These findings prove that the NLC system can provide a significantly higher loading capacity compared to the SLN system.

The released α-mangostin from the NLC system exhibited a high dissolution rate from the early sampling time. The percentage of dissolved α-mangostin from NLC-P-α-M was significantly (~13-fold) higher compared to α-mangostin powder alone. Thus, the NLC system successfully overcame the low solubility issue of α-mangostin in an aqueous medium. This improvement is likely due to the solubilization process mediated by the lipid components in the NLC system. The dissolution profile of NLC-P-α-M correlated well with the increased antioxidant activity of α-mangostin. DPPH scavenging assay results showed that the NLC-P-α-M system at various concentration variations exhibited better inhibition percentages compared to NLC-P or α-mangostin alone. This indicates the synergistic effect between the phenolic content of propolis extract and α-mangostin. However, the antioxidant activity of NLC-P-α-M does not surpass that of ascorbic acid, which was used as a positive control. Both the % inhibition and IC_50_ value of ascorbic acid demonstrated superiority over all test formulations.

## 4. Materials and Methods

### 4.1. Materials

The materials used in this study include distilled water, α-mangostin (Chengdu Biopurify Phytochemicals Ltd., Chengdu, China), propolis (CV Efi Maju Sejahtera, Jakarta, Indonesia), 2,2-diphenyl-1-picrylhydrazyl (DPPH), acetic anhydride, aluminium chloride (AlCl_3_), anhydrous sodium sulfate, chloroform, ethanol 95%, ferric chloride (FeCl_3_), gallic acid, hydrochloric acid (HCl), lecithin, methanol, potassium dihydrogen phosphate (KH_2_PO_4_), potassium acetate (CH_3_CO_2_K), polysorbate 80 (Tween 80^®^), quercetin, sodium carbonate (Na_2_CO_3_), sodium hydroxide (NaOH), Folin–Ciocalteu reagent, and Dragendorff’s reagent (Sigma-Aldrich Co., St. Louis, MO, USA).

### 4.2. Methods

In this study, three main stages were conducted, starting with the preparation of propolis-based nanostructured lipid carriers (NLCs) containing α-mangostin, followed by the characterization of the prepared formulation and concluding with the in vitro evaluation of the antioxidant activity of the formulation.

#### 4.2.1. Propolis-Based Nanostructured Lipid Carrier (NLC) of α-Mangostin Preparation

##### Propolis Extraction

The extraction of propolis was carried out using the maceration method with a weight ratio of raw propolis to extraction solvent of 1:10 [40,41]. The extraction solvent used was 95% ethanol. The maceration of raw propolis was performed in a tightly sealed container at room temperature (25 °C) for 48 h, with agitation every 2 h. The extraction solution was collected every 24 h, followed by replacement with fresh solvent. After the extraction process, the solution was filtered through Whatman No. 1 cellulose filter paper. The obtained liquid extract was stored away from light at 4 °C in an airtight container.

In the next step, the propolis extracted using ethanol was concentrated using a water bath at 70 °C to obtain a concentrated propolis extract ready for formulation. The extraction yield was determined using Formula (1).

Yield (% *w*/*w*) = (viscous extract weight/dry propolis weighted) × 100% (1)

##### Phytochemical Screening of Propolis Extract

To conduct phytochemical analysis, the crude propolis extract was accurately weighed (100 mg) and dissolved in absolute ethanol (40 mL). The same procedure mentioned for phytochemical analysis was then carried out. Subsequently, aliquots of 35 mL from the propolis extract samples, each placed in separate test tubes, were divided into five parts of 3 mL. Additionally, a portion of 10 mL was placed in a beaker. The beaker was then heated in a water bath on a hot plate with continuous stirring until the liquid completely evaporated in order to evaluate the presence of steroids, triterpenoids, and saponins [42].

- Phenols: three drops of an alcohol solution containing ferric chloride (FeCl_3_ 5%) were mixed with the sample. After agitating the mixture, the tube was examined for any color changes or the formation of a significant amount of dark-colored precipitate. The presence of phenols was indicated by hues that ranged from blue to red [42].
- Tannins: the sample was subjected to the addition of three drops of an alcohol-based solution containing ferric chloride (FeCl_3_ 1%). The resulting mixture was then observed for the formation of a precipitate, specifically noting its color. If a bluish dark–green precipitate was observed, it indicated the presence of tannins in the EEP sample [42].
- Flavonoids: carried out by two methods, including alkaline reagent test and Shinod’s test.
  1. Alkaline reagent test: two to three drops of NaOH 10% were introduced into 2 mL of the extract. Initially, a deep yellow color emerged as a result. This color change indicated the presence of flavonoids in the extract [43].
  2. Shinod’s test: the extract was combined with ten drops of concentrated hydrochloric acid (HCl) and a piece of magnesium. As a result, a deep pink color developed, which served as an indication of the presence of flavonoids in the mixture [43].
- Triterpenoids and steroids: the dried residue underwent three extractions with 2 mL of chloroform, and the resulting solution was filtered into a test tube using a cotton-covered funnel with anhydrous sodium sulfate. After filtration, acetic anhydride (1 mL) was added and mixed, followed by the addition of concentrated sulfuric acid (three drops) with agitation. The test tube was then observed for color changes: evanescent blue to permanent green for free steroids, and a range of brown to red for free pentacyclic triterpenoids [42].
- Saponins: the insoluble residue from chloroform was dissolved in 8 mL of distilled water and filtered into a test tube. After vigorous agitation for three minutes, the test tube was checked for the formation of persistent foam indicating the presence of saponin glycosides (saponins) [42].
- Alkaloids: the sample was added by 1 mL of Dragendorff’s reagent. An orange–red precipitate indicates the presence of alkaloids [42].

##### Measurement of Total Flavonoid Levels of Propolis Extract

The total flavonoid content of the propolis extract was measured following the principles of the Dowd method and calculated as equivalents to quercetin [44,45]. Both the propolis extract and quercetin were prepared by dissolving them in a specific amount of 95% ethanol. Aliquots of 1 mL of the propolis extract solution (25–200 ppm) or quercetin solution (25–200 ppm) were mixed with 0.2 mL of a 10% (*w*/*v*) AlCl_3_ solution in methanol, 0.2 mL of 1 M potassium acetate solution, and 5.6 mL of distilled water. The mixture was then incubated for 30 min at room temperature, followed by measuring the absorbance at 415 nm using a using a UV-Vis spectrophotometer (Analytic Jena Specord 200, Jena, Germany). The test results were expressed as a percentage of quercetin equivalents (% *w*/*w*), calculated using Formula (2).

Flavonoid total (% *w*/*w*) = (mass of quercetin obtained/propolis extract weighted) × 100% (2)

##### Measurement of Total Phenol Levels of Propolis Extract

The total phenolic content of the propolis extract was determined using the Folin–Ciocalteu method and calculated as equivalent to gallic acid [44,46]. First, 1 mL of the extract solution (100–500 µg/mL) was mixed with 2.5 mL of 10% (*w*/*v*) Folin–Ciocalteu reagent. After incubating for 5 min, 2 mL of 75% Na_2_CO_3_ solution was added to the mixture and further incubated at 50 °C for 10 min with intermittent agitation. Then, the sample was cooled and its absorbance was measured using a UV-Vis spectrophotometer (Analytic Jena Specord 200, Germany) at a wavelength of 765 nm. The test results were expressed as a percentage of gallic acid equivalents (% *w*/*w*), calculated using Formula (3).

Phenolic total (% *w*/*w*) = (mass of gallic acid obtained/propolis extract weighted) × 100% (3)

##### Propolis-Based Nanostructured Lipid Carrier (NLC-P) Preparation

**Figure 5:**
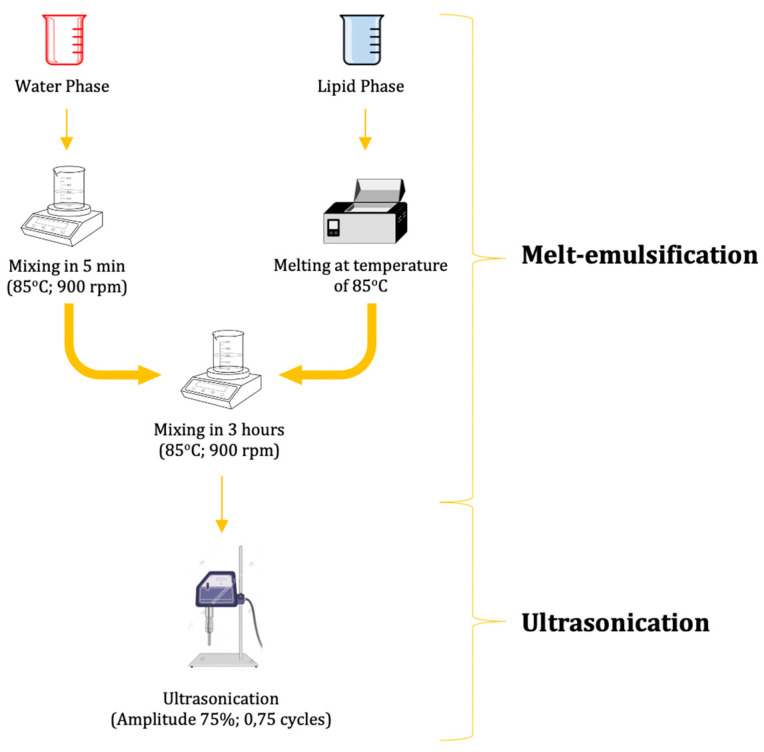
Production of propolis-based nanostructured lipid carrier (NLC) of α-mangostin.

The preparation of propolis-based NLC (NLC-P) was conducted by combining the aqueous phase and lipid phase components listed in Table 4 [33,47]. The NLC-P system was prepared using the melt–emulsification technique combined with ultrasonication, as depicted in Figure 5 [33]. First, the aqueous phase was prepared by mixing polysorbate 80 (Tween 80^®^) and phosphate buffer pH 7.4 at a speed of 900 rpm and a temperature of 85 °C for 5 min. Separately, lecithin and propolis extract were melted to form the oil phase. The oil phase was then added dropwise while continuously stirring at 85 °C until a homogeneous mixture was formed. The mixture was subsequently subjected to sonication for 20 min with an amplitude of 70 and a cycle of 0.5 per second [18,33].

##### α-Mangostin-Loaded Propolis-Based NLC (NLC-P-α-M) Preparation

The preparation of propolis-based NLC with α-mangostin (NLC-P-α-M) was conducted using the same method as the preparation of unloaded propolis-based NLC (NLC-P) (Figure 5), with the lipid phase and aqueous phase compositions listed in Table 4. The difference lies in the preparation of the lipid phase, where α-mangostin was dissolved in 95% ethanol and mixed with melted lecithin and propolis extract. The mixture was then added dropwise to the aqueous phase (polysorbate 80 (Tween 80^®^) and phosphate buffer pH 7.4) while continuously stirring at 85 °C until a homogeneous mixture was formed (for 3 h). Subsequently, the mixture was sonicated at an amplitude of 70 and a cycle of 0.5 per second for 20 min [18,33].

#### 4.2.2. Characterization of α-Mangostin-Loaded Propolis-based NLC

##### Particle Size Analysis (PSA), Zeta Potential (ZP), and Polydispersity Index (PI)

Particle size analysis (PSA) of the NLC formulation was evaluated by determining the z-average score and measuring the polydispersity index (PI) using photon correlation spectroscopy (PCS) in combination with a Zetasizer (NanoSizer 3000, Malvern Instruments, Malvern, UK) at a 90° angle in a cell with a width of 0.01 m at 25.0 ± 0.1 °C. The zeta potential (ZP) of the lipid nanoparticles was determined in a capillary cell using the same instrument, which applies the Helmholtz–Smoluchowski equation to convert the measured particle electrophoretic mobility into zeta potential. The ZP indicates the charge on the surface of the nanoparticles and their physical stability. Prior to analysis, the samples were diluted using phosphate buffer at pH 7.4 at a dilution ratio of 1:100 [48].

##### Transmission Electron Microscopy (TEM) Imaging

The surface morphology of the prepared NLC particles was analyzed using transmission electron microscopy (TEM). A small amount of NLC-P and NLC-P-α-M was dropwise added onto a specially designed copper grid for TEM. The copper grid was then placed in an oven at 45 °C until it was dry. The dried samples were observed using the HT7700 TEM instrument (Hitachi, Tokyo, Japan) [18,49]. The imaging process was performed using a voltage of 100 kV, ×100k magnification, and 0.1 s for video recording. The image was then processed utilizing ImageJ 2.0.

##### Entrapment Efficiency

Drug entrapment efficiency (EE) refers to the percentage of drug trapped within the NLC system compared to the total amount of active substance added. NLC-P-α-M was centrifuged at 5000 rpm for 20 min. The supernatant was then collected and measured at a wavelength of 281 nm using a dual-beam UV-visible spectrophotometer (Analytic Jena Specord 200, Germany) to determine the free (unencapsulated) α-mangostin content [28,50,51]. The entrapment efficiency of α-mangostin in propolis-based NLC was determined using the following equation:

EE (% *w*/*w*) = ((α-mangostin weighted − α-mangostin in supernatant)/α-mangostin weighted) × 100% (4)

##### Dissolution Test

Dissolution testing was performed using a paddle-type dissolution apparatus. PBS (phosphate buffer saline) at pH 7.4 was used as the dissolution medium. A certain number of NLC-P-α-M samples containing an equivalent of 5 mg of α-mangostin as the test sample and 5 mg of α-mangostin powder as the reference were suspended in the dissolution medium. The temperature was maintained at a constant 37 ± 0.5 °C, and stirring was conducted at a speed of 50 rpm. Sample aliquots were collected at 15, 30, 45, 60, 90, 120, 180, and 240 min. The percentage of dissolved α-mangostin was determined by measuring the absorbance using a UV-Vis spectrophotometer (Analytic Jena Specord 200, Germany) at a wavelength of 281 nm [52].

#### 4.2.3. In Vitro Antioxidant Activity Assay of Propolis-Based NLC Formulations

The antioxidant activity of NLC formulations was tested using the DPPH scavenging assay [53]. The DPPH scavenging activity depends on the ability of antioxidant compounds to donate hydrogen to the DPPH radical. Each sample, including NLC-P, NLC-P-α-M, and α-mangostin powder, was prepared by dilution in 95% ethanol at 150, 200, 250, 300, and 350 ppm; 1 mL of 0.2 mM DPPH solution was added to each 1 mL of the sample and mixed. After ten minutes of incubation in the dark, the absorbance was measured using a UV-Vis spectrophotometer (Analytic Jena Specord 200, Germany) at a wavelength (λ) of 517 nm. All tests were performed in triplicate. In addition, a serial dilution of ascorbic acid was prepared as a positive control. The percentage of DPPH radical scavenging was then calculated using the following formula:

Inhibition (%) = ((absorbance of control − absorbance of sample)/absorbance of control) × 100%(5)

## 5. Conclusions

In this research, a propolis-based nanostructured lipid carrier (NLC) of α-mangostin was successfully developed. The carrier has adequate characteristics for nano-scale drug delivery, including particle size, particle size distribution, and entrapment efficiency. The application of NLC in delivering α-mangostin has also been proven to overcome the problem of poor solubility in aqueous media that α-mangostin possesses. The standardized flavonoid and phenol content in propolis extract also demonstrated a synergistic effect with α-mangostin in the NLC system in terms of its antioxidant activity in vitro, as evidenced by an increased inhibition percentage and IC_50_ value in the DPPH scavenging assay. These findings are expected to serve as a basis for utilizing nanotechnology in multidrug delivery and as an initial step for further testing in both in vivo and clinical studies.

## Figures and Tables

**Table 1:** Propolis extract standardization results.

Parameter	Result	Requirement
Yield (%)	48.24	≥11
Flavonoid total (% *w*/*w*)	0.39	≥0.25
Phenolic total (% *w*/*w*)	2.43	≥0.50

**Table 2:** Phytochemical screening results.

Secondary Metabolite(s)	Reagent(s)	Result *
Phenolics	FeCl_3_ 5%	(+)
Tannins	FeCl_3_ 1%	(+)
Flavonoids	Concentrated HCl + Mg	(−)
	NaOH 10%	(+)
Triterpenoids	Concentrated H_2_SO_4_ + acetic anhydride	(+)
Steroids		(−)
Saponins	Agitated then heated	(+)
Alkaloids	Dragendorff	(−)

* (+) indicates the presence of the compound being investigated, and (−) represents the absence of the compound being investigated.

**Table 3:** Characteristics of propolis-based NLC loaded with α-mangostin.

Preparation	PSA (nm)	ZP (mV)	PI	EE (%)
NLC-P	72.7 ± 1.082	−5.100 ± 0.954	0.211 ± 0.028	N/A
NLC-P-α-M	80.3 ± 1.015	−2.767 ± 0.874	0.231 ± 0.028	87.972 ± 0.246

Values are averages ± standard deviation.

**Table 4:** Formulation of propolis-based nanostructured lipid carrier (NLC-P) and α-mangostin-loaded NLC-P.

Component		Unloaded Propolis-Based NLC	α-Mangostin-Loaded Propolis-Based NLC
Lipid Phase	Propolis Extract	0.9 g	0.9 g
	Lecithin	0.24 g	0.24 g
	α-Mangostin *	-	0.01 g
Water Phase	Polysorbate 80 (Tween-80^®^)	2.06 g	2.06 g
	Phosphate Buffer pH 7.4	add 20 mL	add 20 mL

* α-Mangostin was first dissolved in a specific amount of 95% ethanol before being mixed with the lipid phase.

## Data Availability

All data generated or analyzed during this study are included in this published article.
